# Temporal Dynamics of the Sap Microbiome of Grapevine Under High Pierce’s Disease Pressure

**DOI:** 10.3389/fpls.2019.01246

**Published:** 2019-10-04

**Authors:** Elizabeth Deyett, Philippe E. Rolshausen

**Affiliations:** Department of Botany and Plant Sciences, University of California, Riverside, CA, United States

**Keywords:** *Vitis vinifera* L., grapevine, sap, microbiome, Pierce’s disease, *Xylella fastidiosa*, microbiome, xylem

## Abstract

Grapevine is a pillar of the California state economy and agricultural identity. This study provides a comprehensive culture-independent microbiome analysis from the sap of grapevine overtime and in a context of a vascular disease. The vascular system plays a key role by transporting nutrient, water and signals throughout the plant. The negative pressure in the xylem conduits, and low oxygen and nutrient content of its sap make it a unique and underexplored microbial environment. We hypothesized that grapevine hosts in its sap, microbes that have a beneficial impact on plant health by protecting against pathogen attack and supporting key biological processes. To address this hypothesis, we chose a vineyard under high Pierce’s disease (PD). PD is caused by the xylem-dwelling pathogenic bacterium *Xylella fastidiosa*. We selected ten grapevines within this vineyard with a range of disease phenotypes, and monitored them over 2 growing seasons. We sampled each vines at key phenological stages (bloom, veraison, and post-harvest) and used an amplicon metagenomics approach to profile the bacterial (16S -V4) and fungal (ITS) communities of the sap. We identified a core microbiome of the sap composed of seven bacterial (*Streptococcus, Micrococcus, Pseudomonas, Bacteroides, Massilia, Acinetobacter* and *Bacillus*) and five fungal (*Cladosporium, Mycosphaerella, Alternaria, Aureobasidium*, and *Filobasidium*) taxa that were present throughout the growing season. Overall, the sap microbial makeup collected from canes was more similar to the root microbial profile. Alpha diversity metrics indicated a microbial enrichment at bloom and in vines with moderate PD severity suggesting a host-driven microbial response to environmental cues. Beta diversity metrics demonstrated that disease condition and plant phenology impacted microbial community profiles. Our study identified several potential taxonomic targets with antimicrobial and plant growth promoting capabilities that inhabit the grapevine sap and that should be further tested as potential biological control or biofertilizer agents.

## Introduction

The vascular system ensures several key biological functions in plants. The xylem and phloem elements are major vehicles for water and nutrient transportation and signaling routes between the below (i.e., root) and above (i.e., leaf, fruit) ground plant parts. The xylem and phloem saps differ in chemical composition and their profile is also influenced by the plant phenological stages and environmental factors (Andersen et al., 1995; Lucas et al., 2013; Savage et al., 2016). In grapevine, the xylem sap is initially rich in sugars (especially glucose and fructose) and amino acids (especially glutamine) in the spring time right after the dormant season, because of the remobilization of nutrient reserves (e.g. starch) stored in the main permanent structure of the vine (Keller, 2010). This mobilization of nutrients is essential to support vegetative growth at bud-break until leaf becomes energy independent and transition from a sink to a source and export photosynthates and assimilates to the fruit via the phloem sap. To that end, research has focused on how irrigation and fertilization changed sap composition in order to maintain plant hydraulic function at maximum capacity and a balance between vegetative and reproductive growth (Peuke, 2000; El-Razek et al., 2011).

Several biotic and abiotic factors such as freeze damage, drought, and pathogen infection compromise the integrity of the grapevine vascular system and impair plant sap flux (Pouzoulet et al., 2014; Hochberg et al., 2017; Todaro and Dami, 2017). As a result the affected host shows a decrease in vigor and subsequently crop productivity and fruit marketability. One devastating vascular pathogen that plagues the California grapevine industry is Pierce’s Disease (PD), caused by the gram-negative bacterium *Xylella fastidiosa* (Wells et al., 1987). Insect sharpshooters transmit *X. fastidiosa* to the host upon feeding events (Redak et al., 2004). Following infection, the bacterium systemically colonizes susceptible host and multiplies to high titer causing vascular occlusions and a loss of xylem conductivity (Deyett et al., 2019). As a result, grapevine decline rapidly displaying symptoms of canopy dwarfing and thinning as well as leaf scorching and berry raisining (Varela et al., 2001). *In planta*, *X. fastidiosa* resides exclusively in the lumen of the xylem vessel where the negative pressure, low oxygen and nutrient content selects for adapted microorganisms. However, very little is known about the microbial taxa that inhabit the grapevine sap, their population dynamic over a growing season and their interaction with *X. fastidiosa*. Wallis et al. (2013) showed that PD affected the grapevine sap chemical composition but no study has looked at microbial community shifts in response to pathogen infection and colonization of the host and during disease symptoms development.

Grapevine microbiome research has mainly focused on plant organ epiphytes (i.e., berry, leaf and root) because of its importance with grape production and specifically with regards to fruit and foliar diseases management as well as the biological significance of indigenous microbes with the regional signature of a wine (Bokulich et al., 2014; Perazzolli et al., 2014; Zarraonaindia et al., 2015). Identification of the microbial communities inhabiting the grapevine endosphere mocrobiome has been achieved using standard culture-dependent microbial techniques (West et al., 2010; Compant et al., 2011; Baldan et al., 2014; Dissanayake et al., 2018; Kraus et al., 2019). Culture-independent amplicon metagenomic approaches have recently been deployed to improve the microbial profiling of the grapevine woody organs including trunk and cane (Faist et al., 2016; Deyett et al., 2017; Dissanayake et al., 2018). Unraveling the microbiome of the grapevine sap would provide a comprehensive view of the microbes that systemically move throughout the plant and would establish a framework for targeting biological control agents (BCAs) of vascular pathogens or plant growth promoting agents. Identifying BCAs that can control *X. fastidiosa* through direct inhibition or niche displacement and/or commensal and symbiotic organisms that can support the host immune system could become an integrated alternative approach to the current insect vector management strategy. Our research was designed to address this gap in the knowledge by characterizing the bacterial and fungal taxa that shape the xylem sap microbial communities and determining how the host phenology and PD severity impact microbial profile.

## Materials And Methods

### Sap Sample Collection

**Figure 1:**
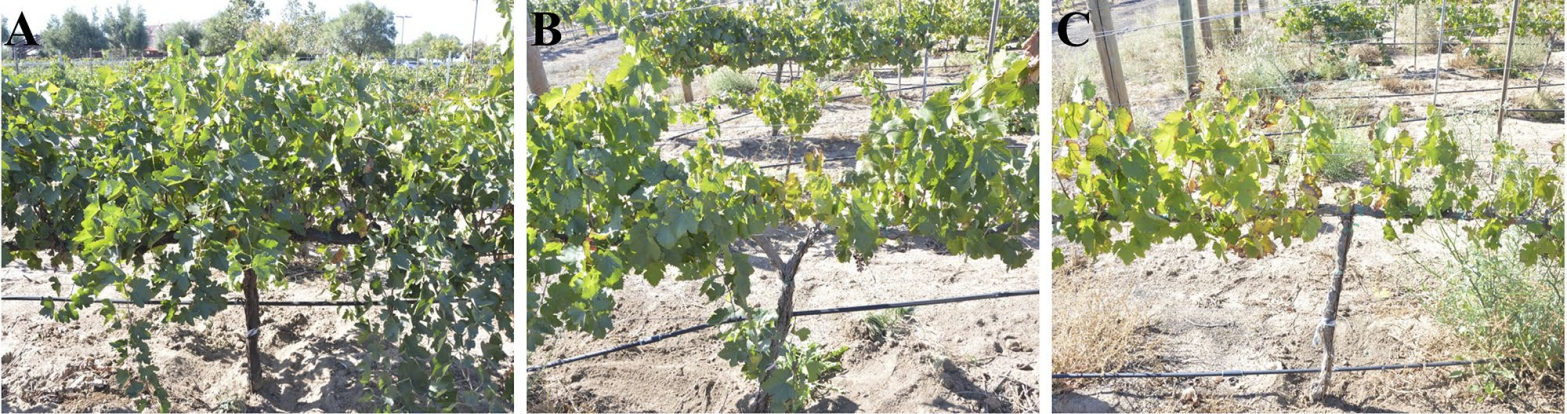
Pierce’s disease (PD) rating scale; **(A)** No symptoms or mild PD symptoms with only a few canes (< 10% of the canopy) showing marginal leaf scorching; **(B)** Moderate PD symptoms with visual dwarfing and thinning on 10-50% of the canopy and/or canes displaying leaf scorching. The affected canes can also show green island and match-stick petioles and berry raisining late in the season. **(C)** Severe PD symptoms with 50–100% of the vine canopy displaying dwarfing, thinning, leaf scorching and wood dieback. The affected canes showed green island and match-stick petioles and berry raisining late in the season.

Shoot samples were collected from a commercial vineyard in Temecula, California, subjected to high insect sharpshooter (the vector of *X. fastidiosa*) pressure. Ten grapevines were selected in 2016 on the basis on PD symptoms, with five healthy vines showing no or mild symptoms and five vines showing intermediate to severe symptoms (**Figure 1**). Canes were later sampled in 2017 and 2018 at bloom (spring), veraison (summer) and post-harvest (fall) from the same grapevines. At each time point, three canes were pruned-off randomly along the grapevine cordons. In addition, grapevines were visually rated for PD symptoms at post-harvest using the same disease rating scale (no/mild, intermediate, or severe PD-symptoms; **Figure 1**). Canes were placed in a cooler with ice packs and brought back to the laboratory. On the same day of the sampling, canes were cut to 40 cm long, inserted in a Scholander pressure chamber (PMS Instruments, Albany OR) and pressurized to 400-550 PSI. Liquid sap was harvested from the end of the cane with a pipette and stored at 4ºC until further processing. To obtain culturable sap microbiome, 20 µl of sap was plated onto each of the following media: lysogeny broth (LB), trypticase soy agar (TSA), and potato dextrose agar (PDA). TSA and LB plates were stored at 28ºC for 3-5 days and PDA plates at room temperature for 7 days. The bacteria and fungi recovered were sub-cultured to obtain single colonies and pure strains, respectively.

### DNA Extraction, PCR Condition, Library Preparation, and Sequencing

The DNA was extracted from 250 µl of sap using the ZymoBIOMICS DNA miniprep kit per manufacturer’s protocol (Zymo Research). The DNA was quantified using a Synergy HTX multi-mode reader (Biotek Instruments, Winooski, VT) for nucleic acid quantification. In addition, the DNA was extracted from pure bacterial colonies and fungal strains using Qiagen blood and tissue kit (Qiagen, Valencia, CA) following the manufacturer’s instructions for cultured cells with an initial extended incubation time of 30 min instead of 10 min.

The bacterial 16S and fungal ITS rRNA region was amplified for each DNA sample using the earth microbiome protocol and primers (http://www.earthmicrobiome.org/). Briefly, primers 515F and 806R with Caporaso barcodes were used for 16S amplification and ITS1f-ITS2 was used for fungal ITS amplification (Caporaso et al., 2010). Each sample was made in a 25 μl PCR reaction using 10 μl of Phusion hot start flex 2x master mix, 0.5 μl of primer (10 μm) and 2 μl of DNA. Thermal cycling parameters for ITS were 94ºC for 1 min; 35 cycles of 94ºC for 30 s, 52ºC for 30 s, 68ºC for 30 s; followed by 68ºC for 10 min. Thermal cycling parameters for 16S amplification were 94ºC for 3 min; 35 cycles of 94ºC for 45 s, 50ºC for 60 s, 72ºC for 90 s; followed by 72ºC for 10 min. Each PCR was accompanied with a negative control to ensure that the mastermix and barcodes were not contaminated. Samples were checked against a 1% agarose gel for visualization of appropriate band size. PCR products were quantified using a Synergy HTX multi-mode reader for nucleic acid quantification. Libraries were prepared by combining equal quantities of DNA from each sample. Libraries were cleaned using AMPure XP PCR purification system (Beckman Coulter) per manufacturer’s protocol. Final concentration of libraries was quantified using qPCR and bioanalyzer before being sequenced on the MiSeq instrument (Illumina, San Diego, USA) using 600-cycle (2X300 paired-end) V3 sequencing kit for fungal reads and the 500-cycle (2x250 paired-end) V2 sequencing kit for 16S at the UCR Genomics Core. Fungal and bacterial sequences were deposited in NCBI under the accession number PRJNA548584.

Culturable microbes were Sanger sequenced using the 27F/1392R primer pair of the 16S bacterial gene (Turner et al., 1999) and ITS1/ITS4 primer pair for fungal ITS region (White et al., 1990). Each sample was made in a 25 μl PCR reaction using 10 μl of Phusion hot start flex 2x master mix, 0.5 μl of each primer (10 μM) and 2 μl of DNA template. Thermal cycling parameters were 94ºC for 2 min; 35 cycles of 94ºC for 1 min, 55ºC for 30 s, 72ºC for 1 min; followed by 72ºC for 5 min. PCR product was visualized on a 1% agarose gel for appropriate band size, cleaned using Qiagen PCR Clean Up kit (Qiagen, Valencia, CA), quantified using Synergy HTX quantifier and submitted for Sanger sequencing at the UCR Genomics Core. Sequences were check for quality before using the NCBI BLAST database for identification.

### Computational Analyses

Raw reads were first filtered for quality using sliding window 5:20 with trimmomatic (Bolger et al., 2014). Reads shorter than 125 bp were removed and low quality reads were filtered out. PhiX reads were then removed from the sequences. Due to the varying lengths of the fungal ITS reads, primers were removed using cutadapt version 1.18 and matching to primer sequences. Libraries were then demultiplexed using QIIME version 1.9.1 (Caporaso et al., 2010). DADA2 version 1.6.0 (Callahan et al., 2016) in R version 3.4.4 was used for the remainder of the processing. Within DADA2, sequences were filtered to contain no ambiguous base calling, and no more than two errors. 16S reads were fixed at lengths for 240 bp. Reads were then de-replicated, and further filtered using the sample inference algorithm and learned error rates within the DADA2 pipeline. Paired end reads were merged using standard arguments. Chimeric sequences were also filtered out. Taxonomy was assigned using the Unite database version 10.10.2017. DADA2 method creates ASV (amplicon sequence variant, similar to operational taxonomic units or OTUs, but is capable of resolving amplicons to a single nucleotide) and thus may make species level identification when 100% of sequences match to reference (Callahan et al., 2016). Bacteria identification was realized using the IDTAXA classification against the SILVA SSU r132 reference database. ClustalW and maximum likelihood parameters were used to create a bacterial tree for UniFrac metrics.

Data were then inputted into the phyloseq package version 1.22.3 (McMurdie and Holmes, 2013). Microbes unidentified at the kingdom and phylum level or classified as plant or other contaminants were removed for both datasets. Phyla that did not occur in at least 5 % of samples were removed as well as singletons, doubletons and taxa that only occurred in one vine. Samples that had too few reads were also removed. For analysis, comparing disease severity, only the post-harvest time points were used. For taxonomy charts, libraries were aggregated to the genus level and transformed to relative abundance. Fungal and bacterial genera were filtered to the 0.1% level. SunburstR version 2.1.0 was utilized to visualize the taxonomy pie charts. Statistical differences between genera in the *Xanthomonadaceae* were computed using Kruskal-Wallis and pairwise comparisons where done with Wilcox pairwise test. Prevalence Venn diagrams was used to identify unique taxa that were more likely associated with the specific grapevine phenological stages at the different sampling time point and with specific disease condition. We set stringent filters with taxa needed to occur in at least 50% of the samples in a given category. UpSetR version 1.3.3 was used to create graphical representation of prevalence Venn diagrams.

Using the number of unique families as a proxy for alpha diversity, mean and standard error were calculated for each time point grouping. Error bars represent standard error of the mean. Statistics on count of family were determined by generalized linear model using Poisson regression and statistical significance on pairwise comparison were conducted through Tukey’s test using the multcomp package version 1.4-8. To look at the difference between groupings, beta-diversity plots were created using weighted UniFrac and PCoA metrics for bacteria and Bray-Curtis dissimilarity matrix and NMDS ordination matrix for fungi using the vegan package version 2.4-5. Adonis test with 999 permutations were run to determine statistical differences among centroids. Pairwise comparisons were calculated with the RVAideMemoire package version 0.9-71. To find differentially abundant microbe associated with season or condition, DeSeq2 was used. Microbes that occurred in more than one sample and in more than one year were used in the analysis. Random Forests supervised machine learning algorithm was applied to the dataset to learn a function that relates predictors (ASVs) to classification, here phonological stage and disease condition (Breiman 2001). Random Forest creates a function using part of the dataset to train and validates that function by classifying the remaining subset of samples. ASVs are assigned with an importance score, which indicates the increase in the functions error rate if the taxa would be removed. In this way, microbes with a high importance score are valuable predictors for a classification. Random forest was carried out using package randomForest version 4.6-14.

## Results

Dada2 analysis revealed that there were 2,875 bacterial amplicon sequence variants (ASVs) and 2,694 fungal ASVs in 68 sap samples (two samples were removed based on poor quality sequences). After removal of contaminants, singletons, doubletons and taxa that did not occur in at least 5% of the samples, there were 168 fungal ASVs and 390 bacterial ASVs. Sap samples that did not have at least 500 reads were removed from the analyses. After filtering, we used a total of 61 samples from the bacterial dataset including 26 from post-harvest, 15 from bloom and 20 from veraison and a total of 65 samples from the fungal dataset including 29 from post-harvest, 16 from bloom, and 20 from veraison.

Phylogeny and relative abundance of fungal and bacterial taxa were represented in a Sunburst plot (http://rpubs.com/edeye001/487241). Results showed that fungal communities were dominated by the *Ascomycota* phylum (∼92%) and the remaining was composed with the phylum *Basidiomycota* (∼8%). *Cladosporium* represented over half (∼56%) of the total fungal genera followed by *Mycosphaerella* (∼16%) and *Alternaria* (∼9%). *Filobasidium* (∼6%) was the major genus among the *Basidiomycota*. The sap culturable mycobiome indicated that *Cladosporium* was also the main fungus recovered and also included the taxa *Filobasidium* and *Mycosphaerella*. The bacterial community composition was more diverse than the fungal community. *Proteobacteria* (∼54%) was the major phylum, followed with *Firmicutes* (∼24%), *Actinobacteria* (∼12%), and *Bacteroidetes* (∼8%). At the genus level an unidentified genus in the *Enterobacteriaceae* family was most abundant (∼19%). *Streptococcus* represented (∼10%) followed with *Bacteroides* (∼6%), *Bacillus* (∼6%), *Acinetobacter* (∼4%) and *Pseudomonas* (∼3%). The culturable bacteria were mostly in the genus *Bacillus*, but *Paenibacillus*, *Virgibacillus*, *Curtobacterium*, and *Micrococcus* were also re-isolated.

**Figure 2:**
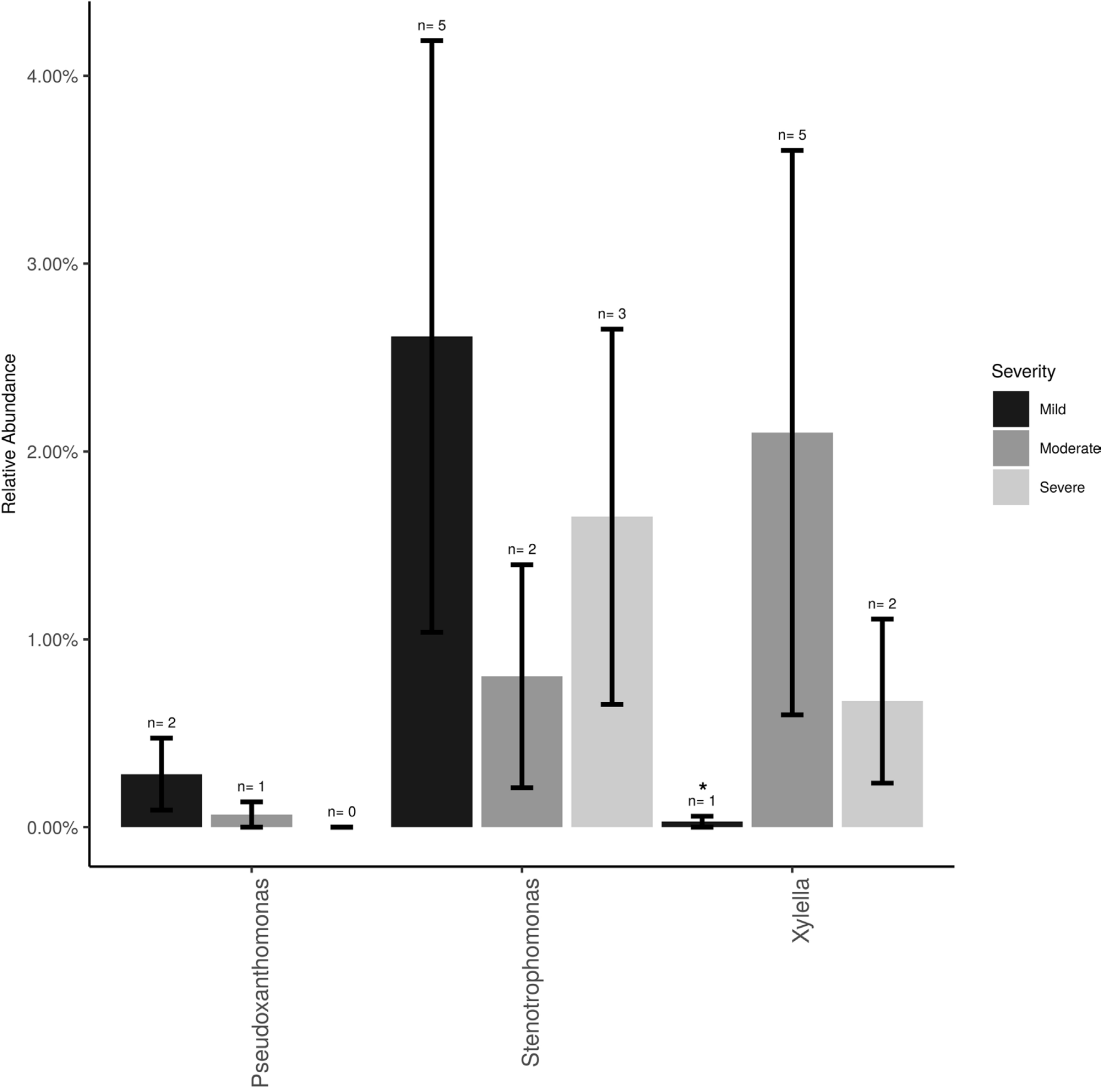
Mean relative abundance and incidence for *Xylella*, *Stenotrophomonas and Pseudoxanthomonas* (Family *Xanthomonadaceae*) in the total sampled grapevines (n = 26) showing no/mild (n = 12), moderate (n = 7) or severe (n = 7) PD symptoms at post-harvest. Error bars represent standard error of the mean. n value above the bar represent the number of vines that tested positive for each bacterium.

The *Xanthomonadaceae* (the family that *X. fastidiosa* belongs to) represented 3% of the overall dataset and was comprised of three genera, *Stenotrophomonas* (∼2%), *Xylella* (∼0.5%), and *Pseudoxanthomonas* (0.5%). *Xylella*, the causal agent of PD, was found at low frequencies and low incidence (11 samples total) and mostly at post-harvest (eight samples of the total 26 post-harvest samples = 31%). *Xylella* was also detected at bloom (one sample) and veraison (two samples). Relative abundance of *Xylella* at the post-harvest time point was only significantly higher (*P* < 0.05) in vines showing moderate PD symptoms compared to healthy vines (i.e., showing mild or no symptoms; **Figure 2**). *Stenotrophomonas* incidence and abundance was the highest in healthy vines although not statistically different from the other disease conditions.

**Figure 3:**
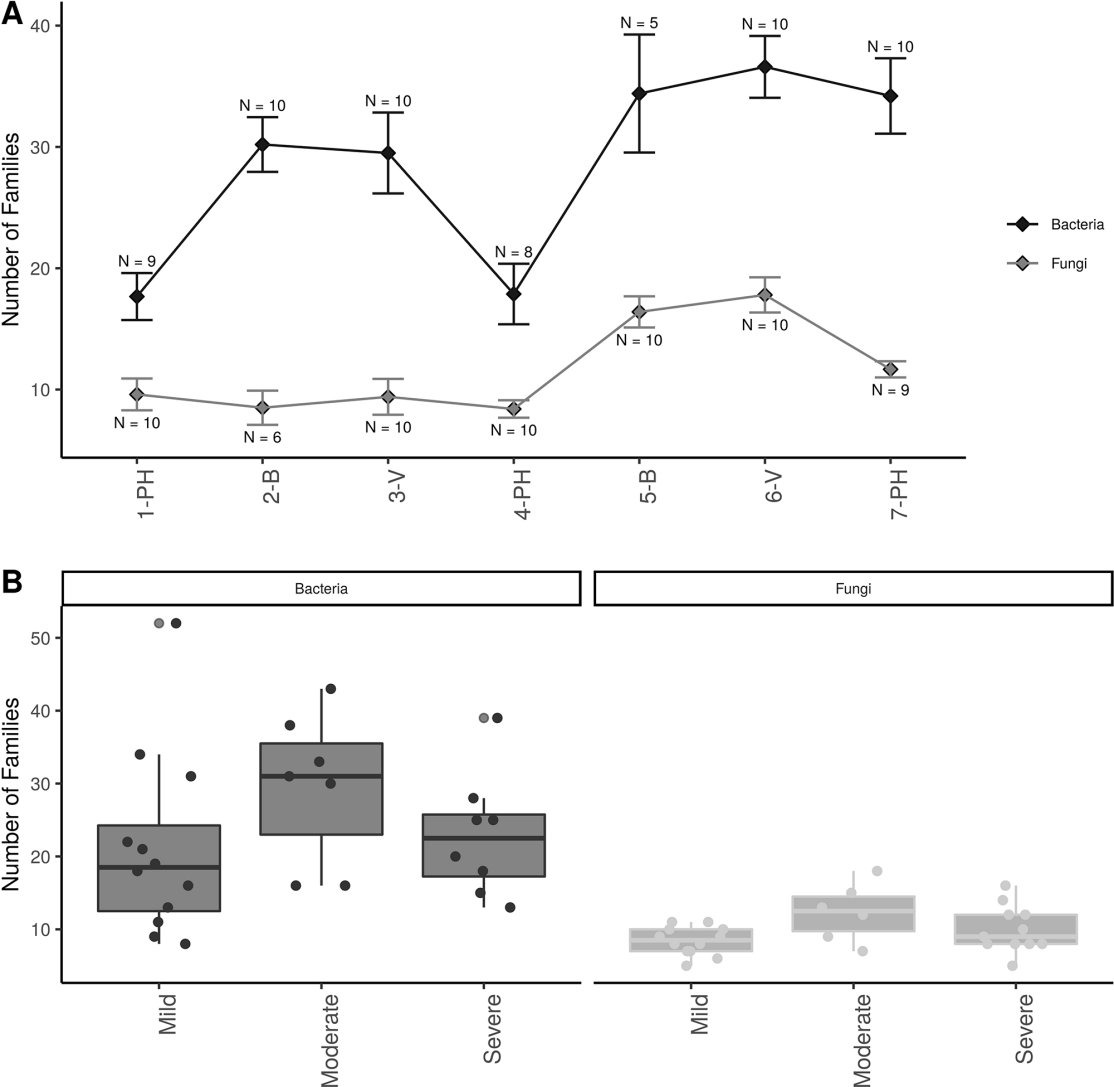
Alpha diversity plots; **(A)** Familial richness in bacterial and fungal sap microbiomes across seasonal timepoints; 1-PH : post-harvest 2016, 2-B : bloom 2017, 3-V: veraison 2017, 4-PH : post-Harvest 2017, 5-B : bloom 2018, 6-V: veraison 2018, and 7-PH : post-harvest 2018. Timepoint scheme is the same across graphs. Error bars represent standard error of the mean. n values represented the number of samples for each time point. **(B)** Number families in bacterial and fungal within different disease conditions at the PH time point. Point represent individual samples.

Alpha-diversity showed that there were approximately twice as many bacterial than fungal families in grapevine sap (**Figure 3A** ). Bacterial family richness increased significantly at post-harvest in aging grapevines between 2016-17 and 2018 (*P* < 0.001). In addition, bloom and veraison time-points displayed significant higher family richness compared to post-harvest in both years for bacteria (*P* < 0.001) and in 2018 for fungi (*P* < 0.01). Moreover, vine condition impacted microbial diversity (**Figure 3B**). Vines categorized as moderately PD symptomatic at post-harvest harbored significantly higher number of bacterial and fungal families compared to mildly symptomatic vines (*P* < 0.05). Severely symptomatic vines also had significantly fewer unique bacterial families compared to moderately symptomatic vines (*P* < 0.05).

**Figure 4:**
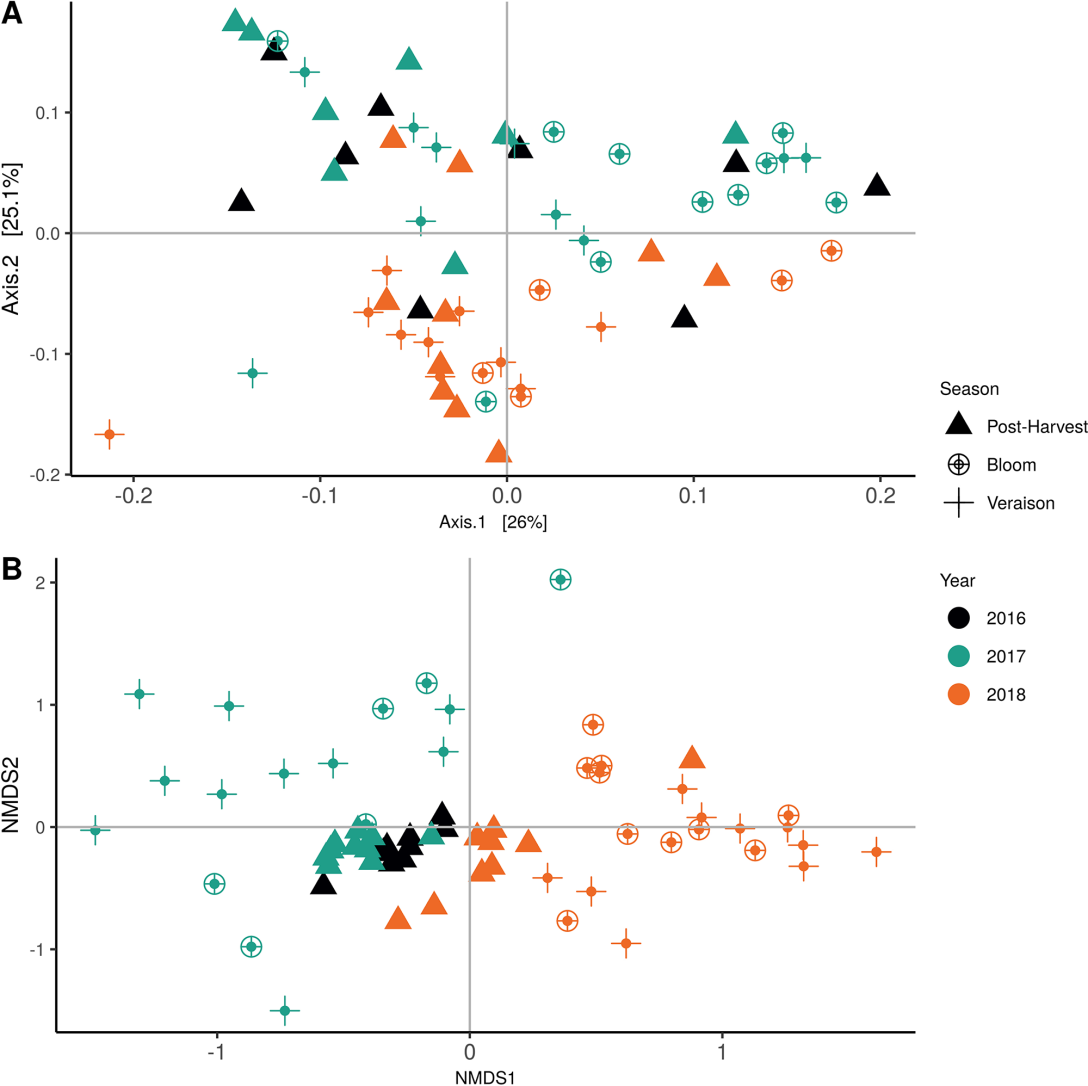
Beta diversity plots for seasonal time points based on: **(A)** Weighted UniFrac distance for bacteria and **(B)** Bray-Curtis dissimilarity for fungi. Points represent one vine community. Point are colored by year, shaped by season.

**Figure 5:**
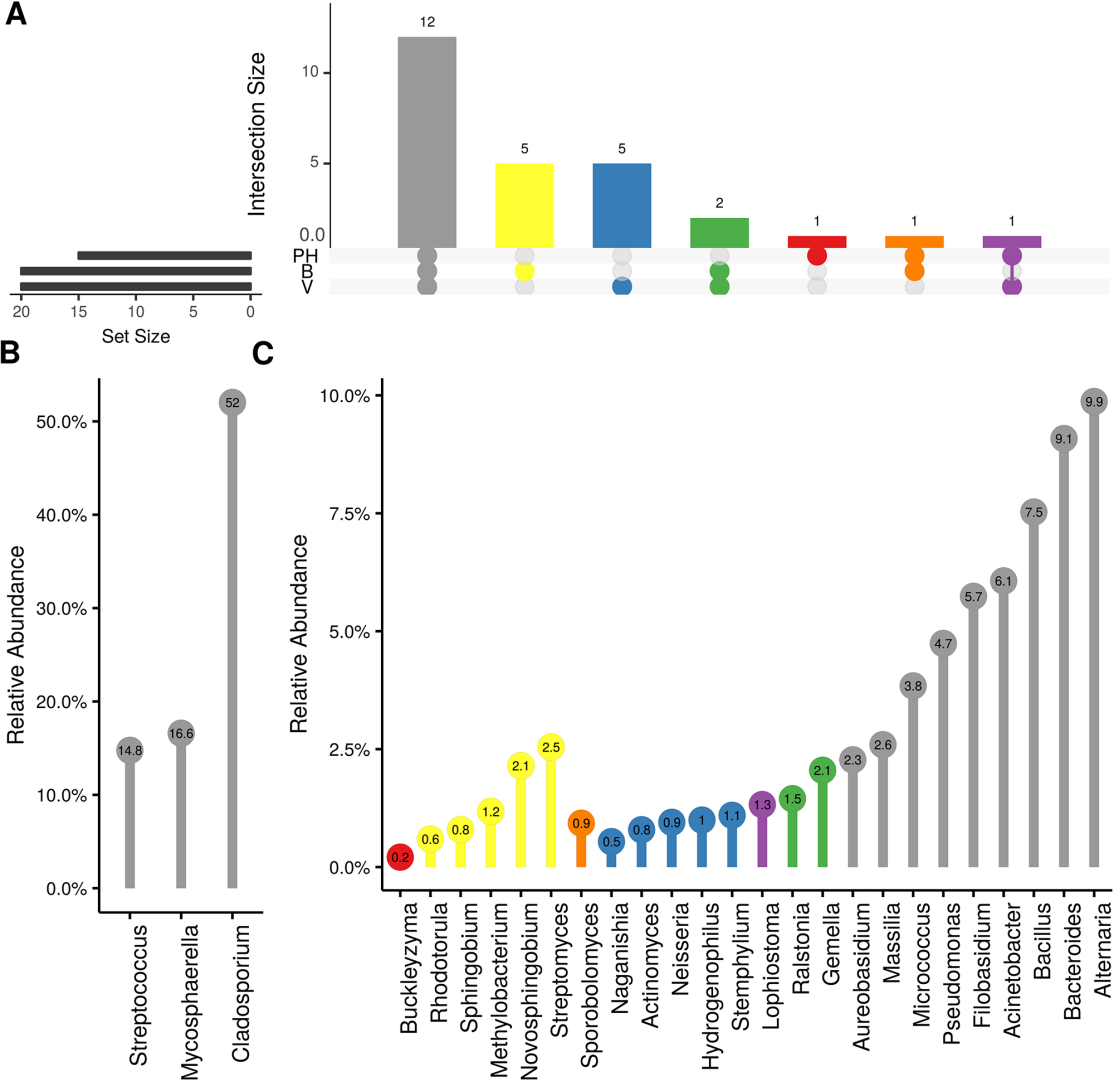
Prevalence Venn diagrams. Genera must occur in 50% of samples for each phenological stage to be considered. **(A)** Quantities of genera intersection between seasonal time points (PH: post-harvest; B: bloom; V: veraison). **(B)** High and **(C)** low relative abundant genera associated with a category. Color scheme is the same as **(A)**.

**Figure 6:**
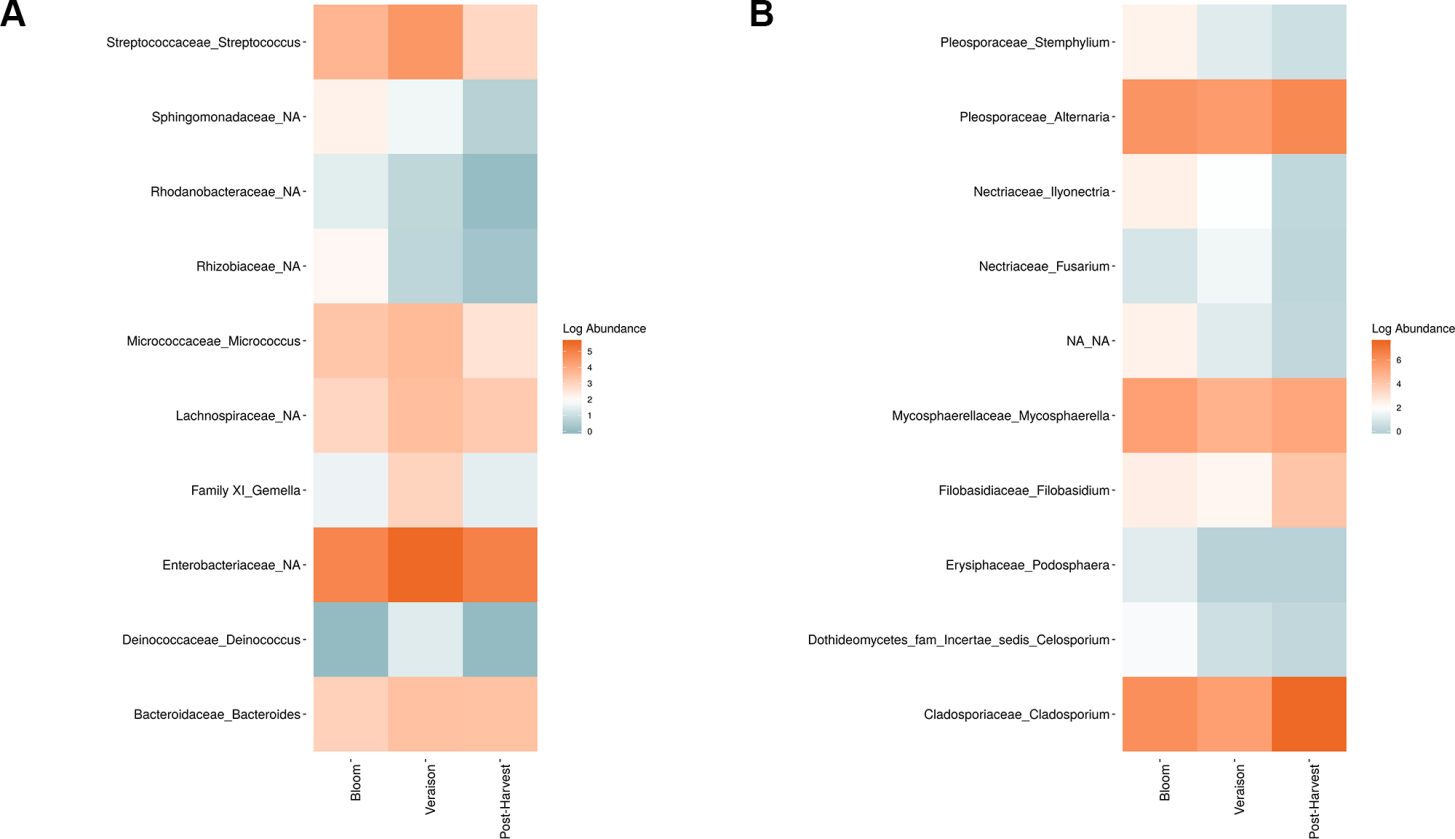
Log abundance of the top 10 random forest important genera for **(A)** Bacteria and **(B)** fungal across seasonal time points.

Weighted UniFrac of bacterial beta-diversity plots (**Figure 4A**) indicated a significant clustering due to the year of sampling (*P* < 0.001) and the time of sampling in a given year (*P* < 0.01). Pairwise PERMANOVA revealed that bloom was distinct from the other two phonological stages (*P* < 0.05) and that 2018 was distinct from 2016 and 2017 (*P* < 0.01). Fungal Bray-Curtis beta-diversity plots (**Figure 4B**) indicated that both year of sampling (*P* < 0.01) and time of sampling (*P* < 0.01) were significantly different in the clustering of fungal communities. There was a strong clustering of the samples collected at post-harvest for all three years in comparison to other sampling year and time. A pairwise PERMANOVA revealed that post-harvest was significantly different to both bloom and veraison (*P* < 0.05) and 2018 was significantly different from both 2017 and 2016, and that 2016 was significantly different than 2017 (*P* < 0.05). Prevalence Venn diagrams indicated that seven bacteria (*Streptococcus, Micrococcus, Pseudomonas, Bacteroides, Massilia, Acinetobacter* and *Bacillus*) and five fungi (*Cladosporium, Mycosphaerella, Alternaria, Aureobasidium*, and *Filobasidium*) were ubiquitous across season at all three plant phenological stages (**Figure 5**). In contrast, other taxa were more likely associated with specific seasonal time points likely contributing for the significant differences in alpha diversity (**Figure 3A**). Hence, *Sphingobium, Novosphingobium, Methylobacterium* and *Streptomyces* were more specific to bloom and *Actinomyces, Neisseria*, and *Hygrogenophilus* to veraison. We used Random Forest analyses to identify predictors (fungal and bacterial taxa) responsible for the clustering in the beta-diversity metrics (**Figure 4**) and identified that the most abundant taxa (*Alternaria, Cladosporium, Filobasidium, Mycosphaerella, Streptococcus, Micrococcus, Bacteroides* and unknown *Enterobacteriaceae*) were the likely drivers (**Figure 6**).

**Figure 7:**
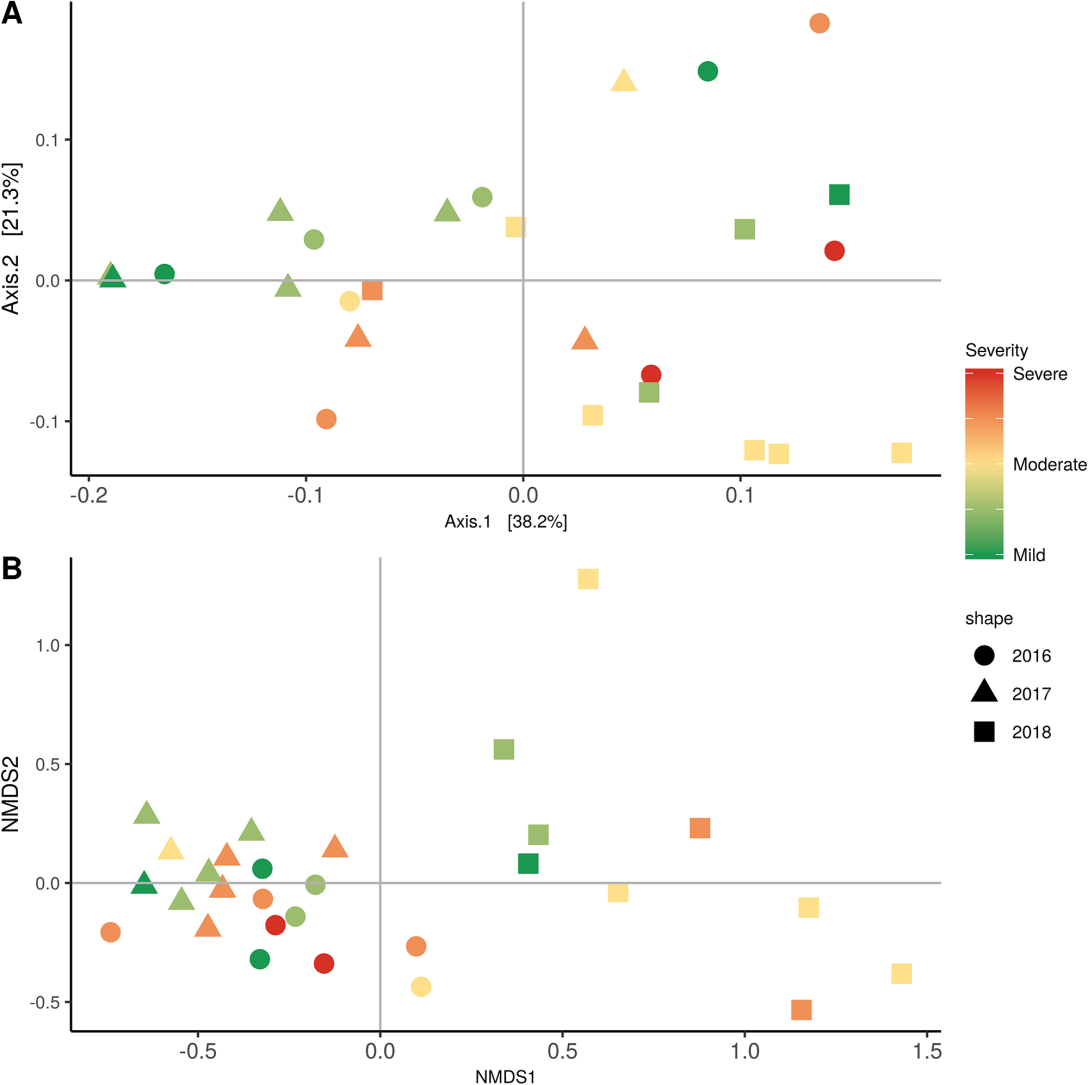
Beta diversity for disease condition plots at post-harvest based on: **(A)** Weighted UniFrac distance for bacteria and **(B)** Bray-Curtis dissimilarity for fungi. Points represent one vine community. Point are colored by PD severity, shaped by year.

**Figure 8:**
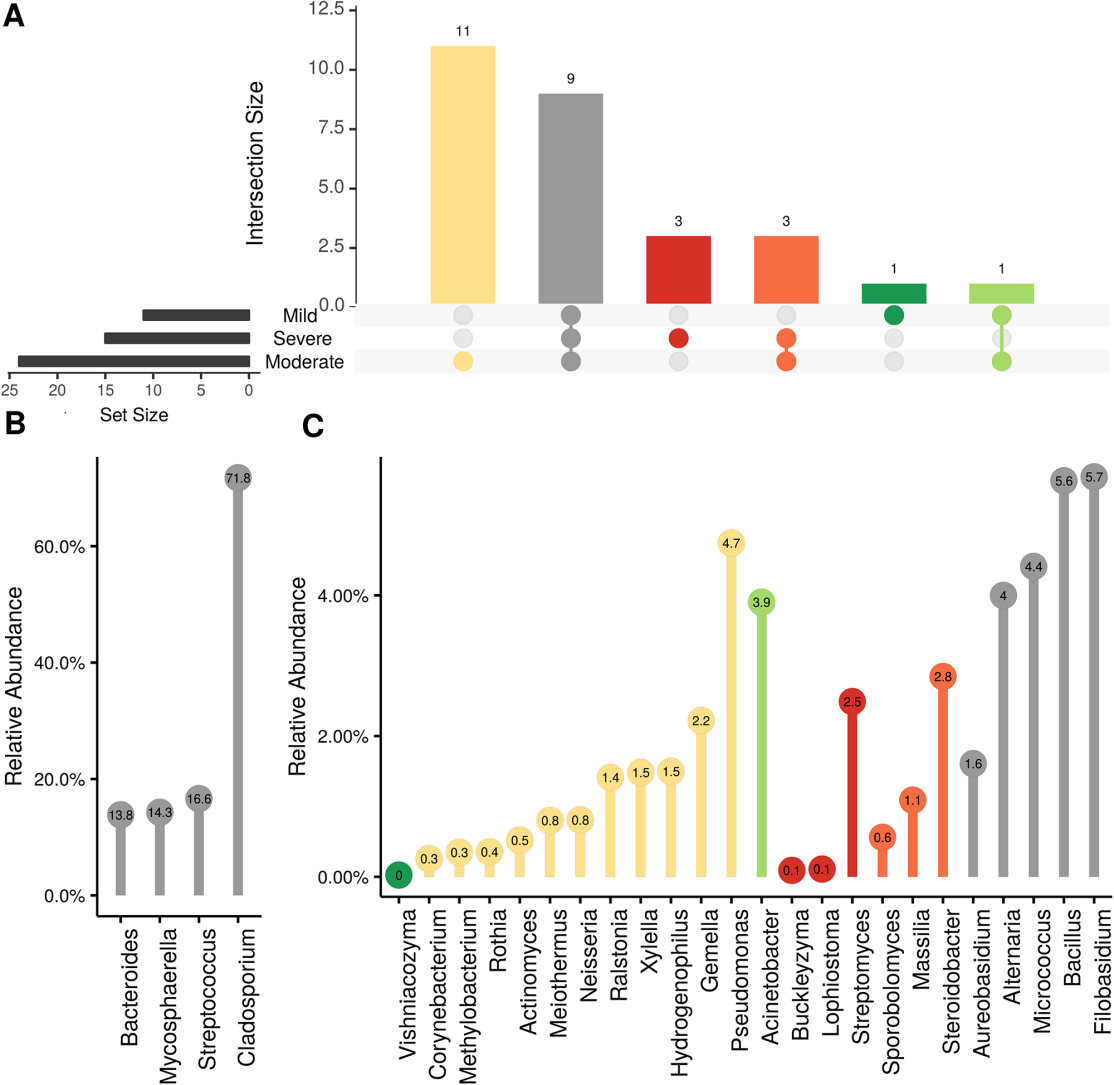
Prevalence Venn diagrams. Genera must occur in 50% of samples for each disease condition at post-harvest to be considered. **(A)** Quantities of genera intersection between disease condition. **(B)** High and **(C)** low relative abundant genera associated with a category. Color scheme is the same as **(A)**.

**Figure 9:**
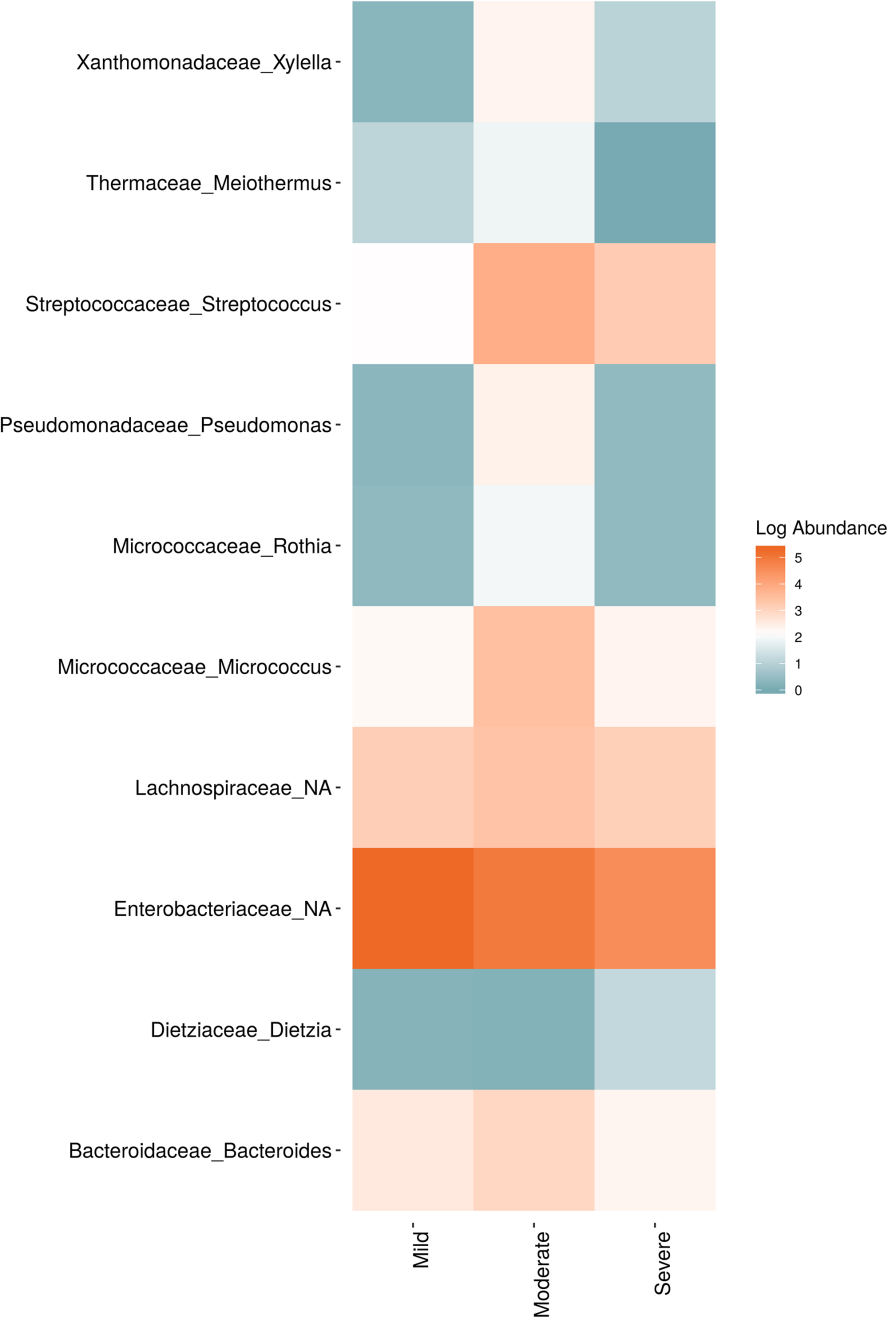
Log abundance of the top 10 random forest important genera for **(A)** Bacteria and **(B)** fungal across disease conditions at post-harvest.

In order to evaluate the impact of disease on microbial communities, we only considered taxa from the post-harvest time points. Beta diversity weighted UniFrac metrics showed that bacterial community clustering was significantly affected by disease severity (*P* < 0.05; **Figure 7A**). In addition, Bray-Curtis metrics indicated that fungal clustering was not affected by disease condition of the vines but was rather attributed to year (*P* < 0.05; **Figure 7B**). Prevalence Venn diagrams showed that several microbes identified in the core microbiome were also found across all grapevines regardless of their disease condition (**Figure 8**). In addition, eleven bacteria (*Corynebacterium, Methylobacterium, Rothia, Actinomyces, Meiothermus, Neisseria, Ralstonia, Xylella, Hydrogenophilus, Gemella*, and *Pseudomonas*) were specific to moderate PD symptoms severity likely contributing to the significant differences in alpha diversity (**Figure 3B**). Random Forest analysis suggested that the clustering in bacterial beta-diversity metrics (**Figure 7A**) was driven by the most abundant bacterial taxa (*Streptococcus, Pseudomonas, Micrococcus, Bacteroides* and unknown *Enterobacteriaceae*), disease condition-specific taxa (*Rothia* and *Meiothermus*) and the pathogen *Xylella* (**Figure 9**).

## Discussion

This culture-independent amplicon-based metagenomics microbiome study establishes a reference framework for the bacterial and fungal communities living in the xylem sap of grapevine and captures temporal microbial shifts during the annual cyclic changes of the host phenology. In addition, it provides insightful information about the impact of the xylem-dwelling pathogen *X. fastidiosa* on the microbiome profile of the grapevine sap.

The grapevine sap mycobiome was most exclusively composed of taxa in the *Ascomycota* in addition to a few yeasts from the phylum *Basidiomycota*. The sap bacteriome was similar to that of other biocompartments with a high abundance of *Proteobacteria*, and lower abundance of *Firmicutes*, *Actinobacteria*, and *Bacteroidetes* (Zarraonaindia et al., 2015; Faist et al., 2016; Deyett et al., 2017; Marasco et al., 2018). Noticeably, no *Acidobacteria* were detected, whereas it was a significant member of the rhizosphere, and root endosphere community in other studies. However, the sap microbiome profile at the phylum level was more similar to the root than the cane, despite the fact that the sap was collected from the later tissue. In addition, we found evidence for the presence of many genera known to be associated with rhizosphere and root endosphere (*Rhizobium*, *Streptomyces*, and *Pseudomonas*) including several pathogenic fungi known to cause root diseases (*Campylocarpon*, *Ilyonectria*, *Fusarium*) (Brum et al., 2012; Cabral et al., 2012). Together these data support the evidence that the microbial makeup of the sap is of belowground origin. This result is in line with a report from another perennial crop (Fausto et al., 2018). However, one should also consider that some organisms, such as *X. fastidiosa,* can also be introduced to the host vascular system by insect feeding and become part of the sap microbiome (Redak et al., 2004; Lopez-Fernandez et al., 2017), while other airborne microbes may enter the plant endosphere through wounds, and plant surfaces *via* stomatas (Compant et al., 2011).

The core microbiome of grapevine sap, that was defined as the microorganisms that occurred in at least 50% of all samples regardless of seasonal time points, was composed of seven bacterial (*Streptococcus, Massilia, Bacteroides, Micrococcus, Pseudomonas, Acinetobacter* and *Bacillus*) and five fungal (*Cladosporium, Mycosphaerella, Alternaria, Aureobasidium*, and *Filobasidium*) taxa. Together, these make up over 51% and 92% of the bacterial and fungal datasets, respectively. *Pseudomonas*, *Bacillus, Micrococcus*, *Acinetobacter, Cladosporium, Aureobasidium Mycosphaerella, Filobasdium* and *Alternaria* have been identified in and/or on many plants organs of grapevine (West et al., 2010; Compant et al., 2011; Baldan et al., 2014; Brysch-Herzberg and Seidel, 2015; Deyett et al., 2017; Dissanayake et al., 2018; Kraus et al., 2019; Nigris et al., 2018). More surprising bacterial taxa included *Massilia*, *Bacteroides* and *Streptococcus*. *Streptococcus* is a facultative-anaerobe bacterium and a well-studied human pathogen. However, it was previously found to be associated with many grapevine biocompartments (soil, root, grape leaf) and across several viticulture areas (Zarraonaindia et al., 2015). *Massilia* is an aerobic bacterium found in a range of habitats (i.e., desert, glacier, water and soils) and was reported to colonize the rhizosphere of plants (Ofek et al., 2012). *Bacteroides* is an anaerobic bacterium that metabolize mono or polysaccharides and is known to colonize the guts of animals and survive in water habitats (Wexler, 2007). One should consider that the more unique bacteria (*Massilia*, *Bacteroides*), might be specific to the viticulture region, because of the water and soil properties. As previously demonstrated for grapevine epiphytes (Bokulich et al., 2014), each viticulture area possess unique indigenous microbial taxa that shape the characteristics of the region, and our data suggests that this paradigm also applies to endophytic microbes. For the other core microbiome taxa, the fact that several of them have been found in association with many grapevine biocompartments both as epiphytes and endophytes, suggest that those could use xylem (and perhaps phloem) as transport routes as previously suggested (Compant et al., 2010; Compant et al., 2011).

Our alpha and beta-diversity metrics showed that the sap microbiome assemblage was shaped by both the plant phenology and disease condition, although the later mainly affected bacterial communities. One should not rule out that the other vascular pathogens also detected in this study (i.e., *Campylocarpon*, *Ilyonectria*, *Fusarium*) may have increased background noise in our analyses. Interestingly we measured a peak in microbial richness during bloom, a key phenological stage for pollination and fertilization. Especially four taxa surfaced at this time point (*Streptomyces, Rhodotula, Sphingobium* and *Novosphingobium*), and those are known to possess plant growth promoting capabilities (El-Tarabily, 2004; Hahm et al., 2012; Seipke et al., 2012; Kim et al., 2019), suggesting that the plant could recruit microbes to participate in key physiological processes. Similarly, vines with moderate PD symptoms displayed higher microbial diversity than in either healthy or severely symptomatic vines. Plants are known to drive microbial assemblage in order to cope with biotic or abiotic stresses and increase environmental fitness (Berendsen et al., 2012; Turner et al., 2013). Perhaps our data indicated a plant-driven microbial response to the pathogen infection. In contrast, in severely symptomatic vines, the toxic environment (e.g., occlusion of xylem vessel with tyloses and decrease of hydraulic conductivity; Deyett et al., 2019) is not conducive to microbial survival.

Asymptomatic grapevines at post-harvest displayed overall a lower pathogen incidence and abundance. The relatively low incidence of *X. fastidiosa* (∼31%) measured in vines exacerbating PD symptoms was comparable to a previously published report (Deyett et al., 2017). These results can be attributed to the sampling design (only 3 canes per vine canopy), the heterogeneous distribution of *X. fastidiosa* in the vine canopy and the occlusion of vessels in severely symptomatic vines make it challenging to recover the bacterium (Deyett et al., 2019). Interestingly, *Stenotrophomonas* abundance was inversely proportional to that of *Xylella* in all three PD rating categories. *Stenotrophomonas is* a closely related bacterium to *Xylella* but is known to benefit grapevine by acting as a biofertilizer and biocontrol agent to nematodes (Aballay et al., 2011; Karagoz et al., 2012). *Pseudomonas* was also found in vines with moderate PD symptoms, although this was a much lower abundance (∼5%) than previously reported (∼80%) from grapevine canes (Deyett et al., 2017; Faist et al., 2016). As previously discussed, *Pseudomonas* likely originates from the roots and colonizes the developing vegetative organs early in the growing season, but unlike most of the other microbial inhabitants of the sap, it appears to have the ability to become established in the lignified structural component of the grapevine vascular system. Deyett et al. (2017) showed that *Pseudomonas* correlated negatively with *Xylella* in PD-affected vines, suggesting that it could be used as a biocontrol agent given its known properties for secreting antimicrobial and plant growth promoting compounds (Khmel et al., 1998; Loper et al., 2012; Pieterse et al., 2014; Gruau et al., 2015). *Paraburkholderia phytofirmans* (formerly known as *Burkholderia phytofirmans*) has also been recognized as a potential biocontrol agent to grapevine PD because it has been identified as an endophyte of grapevine leaf and showed the ability to colonize the grapevine endosphere upon inoculation (root, vascular system and leaf) and prime host disease resistance pathways (Compant et al., 2005;Compant et al., 2008; Lo Piccolo et al., 2010; Baccari et al., 2019). Our data did not support the presence of this specific bacterium in the sap of grapevine but identified several taxa from the family *Burkholderiaceae* (∼5% abundance), including *Massilia* and *Ralstonia* that were a part of the core microbiome and a biomarker for vines showing moderate PD-symptoms, respectively. Finally, *Methylobacterium* also surfaced as a common bacteria in grapevines with moderate PD symptoms. Araujo et al., (2002) described that in the Citrus Variagated Chlorosis (CVC) pathosystem, *Xylella fastidiosa* subsp. *pauca* and *Methylobacterium* acted in synergy and caused an increase in disease severity. Our data support the evidence that *Methylobacterium* is an endophyte of grapevine and that perhaps similar synergistic mechanisms occur in the CVC and PD pathosystems.

This study highlights several potential fungal and bacterial targets with antimicrobial and plant growth promoting properties. Future studies should focus on isolating those potentially beneficial strains and test their biological functions *in vitro* and *in planta* bioassays. The broad host range of *X. fastidiosa* combined with the movement of plant material has led to the introduction of the pathogen to new agricultural areas and made it an emerging global threat (Rapicavoli et al., 2018; Sicard et al., 2018). PD management strategies has relied most exclusively on vector control and the breeding of PD-resistant varieties in grapevine (Krivanek et al., 2006; Daugherty et al., 2015). Few resources have been invested in developing commercial bioproducts that do not select for disease resistance or cause environmental pollution. We hope that this microbiome approach will lay the foundation for innovative research and lead to novel product development and marketing.

## Data Availability Statement

Fungal and bacterial sequences were deposited in NCBI under the accession number PRJNA548584.

## Author Contributions

ED designed the experiment, collected and analyzed the data, and co-wrote the manuscript. PR conceived the original project and research plans and co-wrote the manuscript.

## Funding

We would like to acknowledge the USDA National Institute of Food and Agriculture Hatch Projects 233883 for funding support.

## Conflict of Interest

The authors declare that the research was conducted in the absence of any commercial or financial relationships that could be construed as a potential conflict of interest.
